# Comprehensive characterization of flavor compounds in *goji* berry by HS-SPME-GCMS combined with AntDAS-GCMS for geographical discrimination

**DOI:** 10.1016/j.fochx.2025.102626

**Published:** 2025-06-04

**Authors:** Lu Han, Wan-Ting Zou, Wen-Xin Wang, Long-He Wang, Ping-Ping Liu, Li-Hua Tang, Yi Lv, Yong-Jie Yu, Yuanbin She

**Affiliations:** a College of Pharmacy, Ningxia Medical University, Yinchuan 750004, China; b Zhengzhou Tobacco Research Institute of CNTC, Zhengzhou 450001, China; c Ningxia Food Testing and Research Institute, Yinchuan 750004, China; d College of Chemical Engineering, Zhejiang University of Technology, Hangzhou 310032, China; e Key Laboratory of Ningxia Minority Medicine Modernization, Ministry of Education, Yinchuan 750004, China

**Keywords:** Ningxia *goji*, Flavor compounds, HS-SPME, AntDAS-GCMS, Geographical discrimination

## Abstract

The accurate and comprehensive characterization of flavor compounds in Ningxia *Goji* berry (Lycii Fructus) to provide an accurate geographical discrimination model remains unrealized. Here, a novel strategy that integrates headspace solid-phase microextraction gas chromatography–mass spectrometry (HS-SPME-GC–MS) with our newly developed GC–MS data analysis software, AntDAS-GCMS, was employed for the accurate tracing of samples from typical cultivated zones of China, including Ningxia, Gansu, Qinghai, and Xinjiang province. Parameters of HS-SPME were optimized using the resolved components from AntDAS-GCMS. Information on volatile compounds (VOCs) was collected with GC–MS under the optimized HS-SPME. Raw GC–MS data files were automatically analyzed via AntDAS-GCMS to perform component resolution, time-shift correction and registration, chemometric analysis, and compound identification. A total of 275 components were screened, based on which 30 VOCs were identified. Four chemometric models were evaluated through Monte–Carlo simulation, and the partial least squares-discrimination provided the best geographical discrimination performance with the identified compounds.

## Highlights

• A chemometric model for tracing geographical zones of *Goji* in China was constructed.
• Volatile compounds in Ningxia *Goji* were comprehensively analyzed with AntDAS-GCMS.
• Samples from Ningxia and Gansu were similar but different with Qinghai and Xinjiang.
• PLS-DA model provide the best results compared to the other chemometric models.

## Graphical abstract

**Figure:**
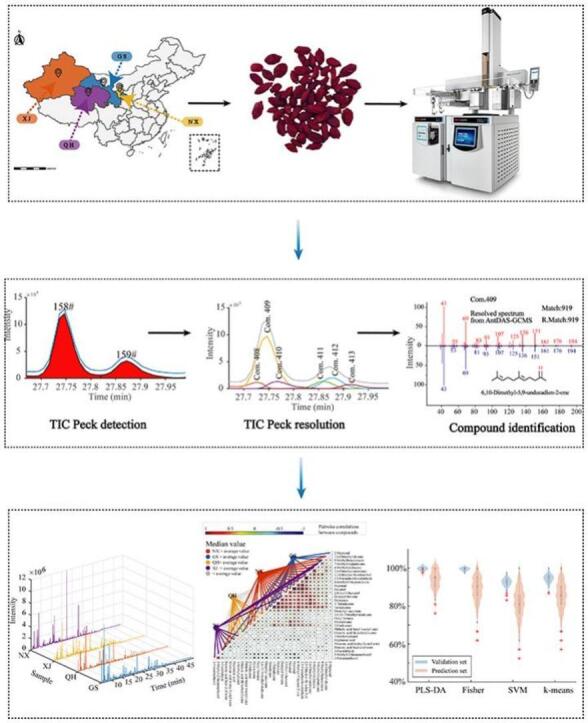

## 1 Introduction

*Goji* berry, which is also well-known as the wolfberry (Lycii Fructus) across the world, refers to the dried fruit of *Lycium barbarum* L.(Cao et al., 2021). This product is recognized as the homology of medicine and food in China and has the biological function of nourishing the liver, improving eyesight, and moistening the lungs (Ma et al., 2022). *Goji* has a long cultivation history in the northwest zone of China, and the Ningxia province has been marked as a genuine production area due to its high quality and medicine function (He et al., 2023; Popović-Djordjević et al., 2023). Ningxia *Goji* berry has become one of the most popular brand of food and health product in China (Popović-Djordjević et al., 2023). Apart from the Ningxia province, the Gansu, Qinghai, and Xinjiang provinces in China can produce *Goji* berry (He et al., 2023), which are frequently transformed into the local market product of Ningxia province and may confuse consumers. Some merchants commonly sell low-cost non-Ningxia *Goji* berry as the Ningxia *Goji* berry to increase their illegal profits. This fraudulent behavior can not only disrupt the market administration but also harm the interests and safety of consumers. Therefore, valid methods for the accurate tracing of the geographical origins of *Goji* berry are urgently needed.

A number of works have been devoted to the geographical discrimination of *Goji* berry using advanced analytical methods, including hyperspectral imaging and elemental and stable isotope fingerprinting. Jiang et al. applied a combination of near-infrared hyperspectral imaging technology and a spectral-image feature fusion convolutional neural network to differentiate the production regions of G*oji* berries (Dong et al., 2022; Jiang et al., 2024). Gong et al. used 49 indices, including stable isotopes, earth elements, soluble amino acids, and saccharides, to identify the regions of these *Goji* fruits (Gong et al., 2022). The technique of chromatography hyphenated with mass spectrometry like liquid chromatography-mass spectrometry (Zhou et al., 2023) and gas chromatography–mass spectrometry (GC–MS) (Ma et al., 2024; Xu et al., 2024) can provide detailed information on chemical compositions and are widely accepted by analysts for geographical tracing of foods (Li et al., 2024). Zhou et al. utilized ultra-fast LC coupled with triple quadrupole time-of-flight MS to distinguish *Goji* berries from different sources (Cheng et al., 2022).

Flavor compounds have been extensively used to characterize analyzed samples, such as foods and herbs. Several research groups employed flavor compounds for the geographical discrimination of *Goji* berry. Peng et al. analyzed volatile compounds (VOCs) in the juice of fresh *Goji* berry in different zones in China (Peng et al., 2024). Zhou et al. utilized the headspace-GC-ion mobility spectrometry to characterize VOCs in *Goji* berry to classify black and red varieties (Zhou et al., 2023). Li et al. employed electronic nose/tongue technology to develop a rapid and accurate strategy for the tracing of *Goji* berry from different zones (Li, Yu, Xu, & Gao, 2017). Even if the current methods provide different solutions for the discrimination of geographical origins and varieties, the comprehensive characterization of VOCs, such as flavors, in *Goji* berry to provide an accurate geographical discrimination remains unsolved.

On the other hand, data analysis of GC–MS presents a challenging task for most researchers in routine analysis. Headspace soild-phase microextraction (HS-SPME) can be used to concentrate massive VOCs for GC–MS but may lead to serious coelution of compounds in the total ion chromatogram (TIC) (Fan et al., 2022; Smirnov, Jia, Walker, Jones, & Du, 2017), which, without a proper treatment, may lead to invalid conclusions. To address this problem, our research group developed a novel GC–MS data analysis toolbox, AntDAS-GCMS, to automatically perform TIC peak detection, coeluted component resolution, component registration, compound screening, and identification (Ma et al., 2024; Ma et al., 2023). In addition, statistical analysis and chemometric classification, including analysis of variance (ANOVA) and partial least square discrimination analysis (PLS-DA) were supported in AntDAS-GCMS. Briefly, AntDAS-GCMS may provide a new solution for VOC analysis in *Goji* berry.

In this work, flavor compounds of *Goji* berry were comprehensively studied via HS-SPME-GCMS (Liang, Wu, Lu, & Li, 2024) coupled with AntDAS-GCMS (Zhang et al., 2020) to provide a geographical discrimination model for the accurate recognition of Ningxia *Goji* berry in the local market. An instrument optimization strategy was developed for HS-SPME-GCMS to comprehensively collect information on VOCs. The optimized HS-SPME-GCMS was used to analyze the *Goji* berry from Ningxia, Gansu, Qinghai, and Xinjiang provinces in China. Raw data files were imported into AntDAS-GCMS to perform component resolution, alignment, and registration. Flavors that showed significant differences were screened via ANOVA and identified using the National Institute of Standards and Technology (NIST) and an in-house library. Finally, various chemometric models, including PLS-DA, Fisher discrimination, support vector machine (SVM), and *k*-means clustering, were conducted to trace the geographical origins of *Goji* berry, and their performances were confirmed via Monte–Carlo simulation.

## 2 Materials and methods

### 2.1 Sample collection

A total of 104 *Goji* berry samples were collected from four cultivated regions, including Ningxia, Gansu, Qinghai, and Xinjiang provinces in China (**Figs. S1)**. Specifically, 30, 25, 24, 25, samples were obtained from Ningxia, Gansu, Qinghai, and Xinjiang respectively. **Table S1** lists the specific geographical locations of the 104 *Goji* berry samples. The collected *Goji* berry samples were oven dried at the constant temperature of 40 °C for 48 h, ground to powder, and stored in a dry and cool place until analysis.

### 2.2 Flavor captured by HS-SPME

Flavors were captured and analyzed using a PAL RSI multifunctional SPME system (Switzerland). A DVB/PDMS-65 μm (SPME) was used to concentrate flavors released from *Goji* berry. A total of 2.0 g *Goji* berry sample was precisely weighted and placed in a HS flask, which was heated up to 80 °C and maintained for 25 min to release the VOCs within the sample. After a 10 min capture through SPME at room temperature, the collected VOCs were analyzed using an Agilent 8890-5977B GC–MS.

### 2.3 GC–MS analysis

An Agilent DB-5MS capillary column (60 m × 250 μm, 0.25 μm film thickness) was used to separate captured compounds via HS-SPME. The oven temperature was initialized at 70 °C for 2 min and then increased to 160 °C at the rate of 4 °C/min. After a maintenance of 2 min, the temperature was increased to 280 °C at 10 °C/min and was held on for 10 min. A postrun was employed at 300 °C for 10 min. Helium (99.999 %) was used as the carrier gas at a flow rate of 1 mL/min, and the split ratio was set as 5:1.

An EI ion source with an ionization voltage at 70 eV and a transfer line temperature of 280 °C was used. The ion source and quadrupole had temperatures of 230 °C and 150 °C, respectively. Data acquisition was carried out in full scanning mode withing a mass range of 33–500 Da. The scanning speed was 781 u/s.

### 2.4 Chemometrics analysis

Raw data files were imported into our AntDAS-GCMS for automatic TIC peak detection, component resolution, time-shift alignment, and peak registration. Finally, a compound registration table was obtained, and it can be used for the subsequent chemometric analyses, including compound screening and geographical origin discrimination (Zhao et al., 2022). In addition, AntDAS-GCMS (http://www.pmdb.org.cn/antdasgcms.) can export an MSP file containing all screened compounds from the NIST for compound identification.

ANOVA was used to screen resolved compounds that revealed significant differences among geographical sources. Supervised discriminant methods, including PLS-DA, Fisher discrimination, SVM, and *k*-means, were used to discriminate samples from different zones. All data analyses were performed using Matlab.

## 3 Results and discussion

### 3.1 Performance investigation for AntDAS-GCMS

**Fig. 1:**
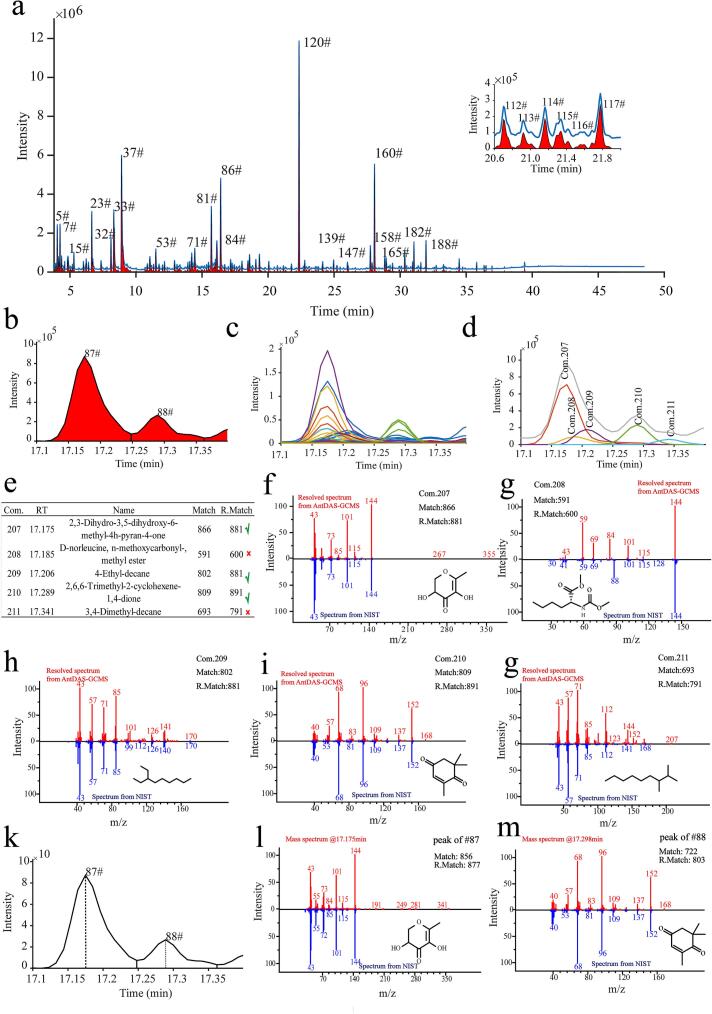
Graphical illustration of volatile compound resolution and identification in AntDAS-GCMS. (a) TIC peaks extracted from a *Goji* sample. (b-d) TIC peak deconvolution procedure in AntDAS-GCMS. Two TIC peaks (b) and ion chromatograms under the elution range (c). (d) Five components were resolved from the two TIC peaks. (e) Compound identification results for the Five components. (f-g) Resolved mass spectra of Five identified compounds. (k-m) The classic manual compound identification was shown.

The performance of AntDAS-GCMS on the retrieval of compounds in GS-MS data files was investigated (Ma et al., 2023). Fig. 1 graphically illustrates the analysis procedure for the flavors in the *Goji* berry sample through AntDAS-GCMS. Fig. 1a depicts the extracted TIC peaks in a TIC. A total of 240 TIC peaks were extracted and labeled with numeric values through AntDAS-GCMS. Although the TIC peaks with a high abundance obtained acceptable separation on the TIC, the low-abundance compounds overlapped without a baseline separation. The inset plots in Fig. 1a provide a detailed visualization of the detected TIC in one elution zone, where the compounds were coeluted seriously. The coelution problem in the TIC can lead to incorrect estimation of qualitative and quantitative results for the analyzed compounds, which need to be precisely analyzed before statistical analysis.

Figs. 1b-1d show TIC peak resolution procedure in detail in AntDAS-GCMS. Two TIC peaks were detected (87# and 88#, Fig. 1b) with peak detection agorithm. Fig. 1c depicts the extracted ion chromatograms (EICs) involved in the elution range of the two TIC peaks. The EICs were arranged into a resolution matrix to perform component resolution with multivariate curve resolution (Tauler, 2020). Five clusters were found from the EICs on the basis of elution profiles. Finally, five components were retrieved (Fig. 1d**)**. Three resolved components, including Com.207, Com.208, and Com.209, were eluted under the TIC peak of 87#. Similarly, two resolved components, including Com.210 and Com.211, were observed within the elution range of the TIC peak of 88#. The resolved mass spectra associated with resolved were imported into NIST for compound identification and the results are shown in Fig. 1e. The mass spectra of resolved compounds were shown in Figs. 1**f-**1**g**, together with the ones in the NIST. The quality of identification were evaluated with both match factor (MF) and revised match factor (RMF), which were calculated as a combination of a item of dotted product of weighted mass spectra and a composite item (Stein & Scott, 1994). Here, the thresholds of MF and RMF from NIST above 800 were used as the acceptability criteria. It can be found that three compounds were identified (Fig. 1e). The classic manual compound identification was shown in Figs. 1k-1m, where analysts utilized the mass spectra at peak apexes of the two TIC peaks for compound identification. It can be found that only a compound can be accepted, whose MF and RMF were above 800. A comparison suggested that both the number of identified compounds and the quality of MF and RMF can be greatly improved by AntDAS-GCMS. The results indicate the use of AntDAS-GCMS for compound detection and resolution in the flavor analysis of *Goji* berry.

### 3.2 Optimization of HS-SPME-GCMS with AntDAS-GCMS

#### 3.2.1 Optimization of SPME Fiber

Fiber coating materials exhibit various affinities for analyzed compounds. The selection of an appropriate SPME fiber can greatly enhance the coverage of analyzed compounds within a sample (Liu et al., 2024). The SPME fiber materials were qualitatively studied via AntDAS-GCMS to reveal their effects on compound adsorption. The performance of SPME was evaluated by resolving the TIC peaks with AntDAS-GCMS. The number of resolved components and the total peak area of resolved components were in for the evaluation.

In this study, four types of SPME fiber were evaluated, PDMS-100 μm, DVB/Carbon WR/PDMS-80 μm (50 μm/30 μm), carbon WR/PDMS-95 μm, and DVB/PDMS-65 μm. The collected GC–MS data were imported into AntDAS-GCMS to perform TIC peak detection and component resolution for a fair comparison among SPME fibers. **Figs. S2a** and **S2b** depict the TICs and resolved components, respectively. The compounds adsorbed by different SPME fibers varied. The results corresponding to the total peak area of resolved components suggest that carbon WR/PDMS-95 μm and DVB/PDMS-65 μm fibers yielded larger values than the other two fibers. A further inspection unveiled that DVB/PDMS-65 μm provided better results than the carbon WR/PDMS-95 μm fiber in terms of the number of resolved components. Thus, the DVB/PDMS-65 μm fiber head was selected for the subsequent experiment optimization.

#### 3.2.2 Optimization of the sample amount and adsorption time

Given the sensitivity of GC–MS and the concentration capability of SPME, a certain sample amount was then optimized to avoid the overloading problem. Meanwhile, the maximum number of released compounds from the *Goji* berry was determined. The DVB/PDMS-65 μm SPME was used to concentrate the released flavor compounds at various sample amounts (1.0, 1.5, 2.0, and 2.5 g). The TICs are depicted in **Fig. S3a**, where depicted revealed a slight difference. **Fig. S3b** provides the component resolution results of AntDAS-GCMS. The number of resolved components increased to the largest value at the sample amount of 2.0 g. On the contrary, the total peak area suggested a decrement tendency with the further increment in the sample amount. In this case, the sample amount of 2.0 g was selected for the analysis.

The adsorption time of SPME was then optimized with six sampling time points, i.e., 20, 25, 30, 35, 40, and 50 min. **Figs. S4a** and **S4b** present the results of TICs and resolved components from AntDAS-GCMS, respectively. **Fig. S4a** indicates that TICs of different adsorption times were similar to each other. The resolved components in **Fig. S4b** were subjected to quantitative evaluation, and adsorption times of 25 and 30 min provided larger values than the others for the number of resolved components. However, the resolved compounds covered a lower total peak area under 30 min than under 25 min. Here, the adsorption time of 25 min was selected for further analysis.

#### 3.2.3 Optimization of sample heating temperature

The heating temperature of samples in the HS bottle was optimized, and four heating temperatures (60 °C, 70 °C, 80 °C, and 90 °C) were investigated. The TICs in **Fig. S5a** suggest that TICs under different temperatures were similar to each other. However, The component resolution results of **Fig. S5b** indicate that there is a little difference under various heating temperatures. The component resolution results of **Fig. S5b** indicate that the number of resolved components and total peak area gradually increased with the increment to the largest value at 80 °C, and then both the number of resolved components and total peak area decreased. The results suggest that VOCs can be greatly released at 80 °C. However, a further increment in the heating temperature can not only reduce the adsorption efficiency of SPME, but may also greatly enhance additional chemical reactions like Maillard reaction. In this work, samples in the HS bottle were heated at a temperature of 80 °C.

### 3.3 Analysis of VOCs in goji berry

#### 3.3.1 Component screening via AntDAS-GCMS

**Fig. 2:**
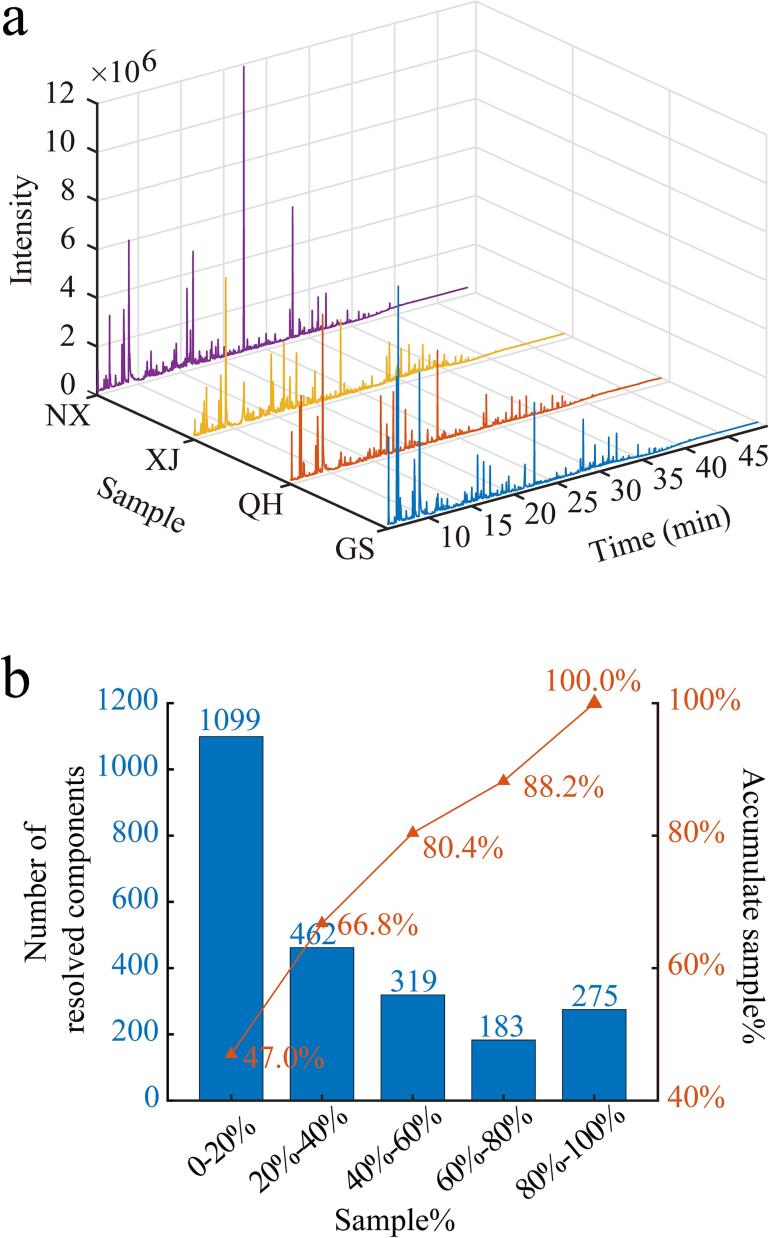
(a) Typical TICs of *Goji* samples from different zones. (b) Statistical analysis of registered components from AntDAS-GCMS. NX, GS, QH, and XJ represent Ningxia, Gansu, Qinghai, and Xinjiang provinces, respectively.

With the optimized HS-SPME, the collected samples were analyzed, and the typical TICs of *Goji* berries from four producing zones are depicted in Fig. 2a. A majority of adsorbed compounds from HS-SPME were distributed within the first 40 min of the TICs. The TICs from various zones exhibited a slight difference in their peaks. The TIC intensity observed in Ningxia province is higher than those in the remaining three zones. The GC–MS raw data of collected *Goji* berry samples were analyzed via AntDAS-GCMS to register a component table of 2338 × 104, where 2338 and 104 represent the number of registered components and the number of samples, respectively. Further analysis of the registered components indicated that 275 components can be detected by 80 % samples (Fig. 2b), and they were used for the subsequent differential compound screening, identification, and geographical discrimination.

**Fig. 3:**
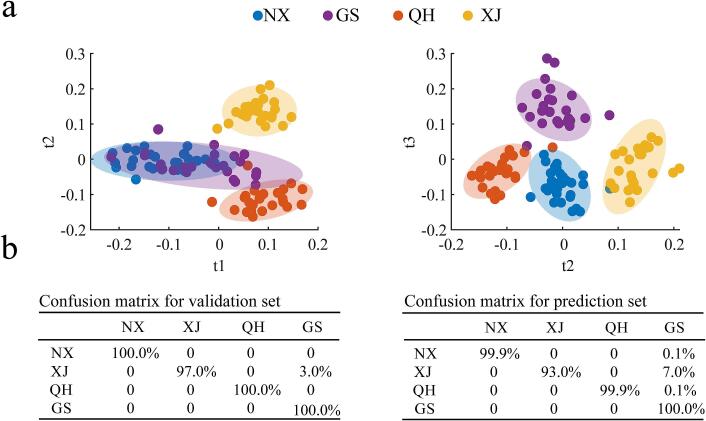
(a) PLS-DA results based on the screened 275 components present in at least 80 % of the samples. (b) Results on validation and and prediction sets after a 10,000 Monte-Carlo simulation. NX, GS, QH, and XJ represent Ningxia, Gansu, Qinghai, and Xinjiang provinces, respectively.

Supervised PLS-DA was used to visualize the differences in *Goji* berries of various origins using the 275 components. Fig. 3a shows the sample distribution on the first two latent variables of the PLS-DA model and the distinct separation of *Goji* berries from Qinghai and Xinjiang provinces. By contrast, the berries from Ningxia and Gansu provinces displayed a serious overlap, this phenomenon may be attributed to inherent similarities in the geographical locations of cultivation, climatic conditions, and agricultural practices between Gansu and Ningxia *Goji* berries. When we focused on the sample distribution characteristics on the second and third latent variables of PLS-DA, the samples from the four zones can be separated clearly, especially for the overlapped zones of Ningxia and Gansu provinces. The sample clustering results in Fig. 3a imply that samples from different zones may be separated via PLS-DA with multiple latent variables.

The classification and prediction capabilities of PLS-DA was also investigated through Monte–Carlo simulation. A total of 80 % of the samples within the analyzed dataset were randomly selected as a validation set to construct a PLS-DA model for prediction of the locations of the remaining 20 % unknown samples. Monte–Carlo simulation was performed in 10,000 times in this work, and the results are shown in Fig. 3b. After a 10,000 simulation, the validation samples obtained an accuracy of 100 %. A more important issue is the prediction accuracy of unknown samples. Findings suggest a slightly different prediction accuracy for samples from various zones. Specifically, Ningxia, Gansu, and Qinghai obtained prediction accuracies reaching 99.9 %, which is higher than that of Xinjiang (93.0 %). The confusion matrix suggested that a small part of samples from Ningxia, Xinjiang, and Qinghai provinces were mis-classified into the Gansu province. The prediction results imply that the combination of VOCs with chemometric PLS-DA model may result in the satisfactory classification of *Goji* berries from different geographical origins.

The 275 components were imported into NIST for compound identification for in-depth comprehension of compounds that may be valuable for geographical discrimination. Compounds with MF > 0.7 and retention index tolerance <50 were selected in this work. Finally, 30 compounds were successfully identified, and their information is provided in Table 1. The 30 compounds can be roughly divided into eight groups based on their chemical structures, including alkanes (9 compounds), esters (7 compounds), aldehydes (6 compounds), alcohols (4 compounds), olefins (1 compound), ketones (1 compound), phenols (1 compound), and acids (1 compound).

**Table 1:** Detailed information of the matching 30 compounds.

RT	ID	Cal RI	Name	CAS	Formula	MF	RI-nbLib	RI-Dist	PLS-DA VIP	Class	Flavor
6.65	140	802	Hexanal^a,b^	66–25-1	C_6_H_12_O	0.9522	800	2	0.85	aldehydes	fruit flavor
7.36	179	828	3-Furaldehyde^a,b,c^	498–60-2	C_5_H_4_O_2_	0.9528	832	4	0.81	aldehydes	woody flavor
7.72	192	841	3-Furanmethanol^c^	4412-91-3	C_5_H_6_O_2_	0.8260	835	6	1.14	alcohols	/
9.02	261	889	Heptanal^a,b^	111–71-7	C_7_H_14_O	0.8870	901	12	0.73	aldehydes	fruit flavor
10.41	339	941	5-Methyl-2-furanmethanol^c^	3857-25-8	C_6_H_8_O_2_	0.9297	958	17	1.00	alcohols	burnt sweet flavor
10.69	352	951	2-Heptenal^b^	57,266–86-1	C_7_H_12_O	0.8694	958	7	1.23	aldehydes	/
11.50	390	981	6-Methyl-5-hepten-2-one^a,b^	110–93-0	C_8_H_14_O	0.8467	986	5	0.61	ketones	fruit flavor
12.95	499	1054	2-Ethyl-1-hexanol^a,b^	104–76-7	C_8_H_18_O	0.9018	1030	24	0.66	alcohols	flower flavor
13.00	504	1057	Dimethyl succinate^a,b^	106–65-0	C_6_H_10_O_4_	0.8374	1030	27	1.55	esters	fruit flavor
13.12	510	1064	2,5-Furandicarboxaldehyde^a^	823–82-5	C_6_H_4_O_3_	0.8804	1076	12	1.21	aldehydes	/
13.30	523	1075	3-Methyl-phenol^c,d^	108–39-4	C_7_H_8_O	0.8261	1075	0	0.71	phenols	woody flavor
14.16	587	1124	Heptanoic acid^c,d^	111–14-8	C_7_H_14_O_2_	0.8143	1078	46	1.20	acids	oil flavor
14.42	600	1138	Octyl formate^d^	112–32-3	C_9_H_18_O_2_	0.9228	1114	24	1.04	esters	fruit flavor
14.78	624	1159	3,6-Dimethyl-decane^a,b^	17,312–53-7	C_12_H_26_	0.8320	1129	30	0.63	alkanes	/
15.50	676	1201	Dodecane^a,b^	112–40-3	C_12_H_26_	0.9206	1200	1	0.80	alkanes	/
16.02	698	1230	2,6-Dimethyl-undecane^a,b^	17,301–23-4	C_13_H_28_	0.8716	1210	20	0.74	alkanes	/
16.73	735	1269	4-Methyl-dodecane^a,c^	6117-97-1	C_13_H_28_	0.8201	1259	10	1.01	alkanes	/
17.45	788	1309	2,6,11-Trimethyl-dodecane^a,d^	31,295–56-4	C_15_H_32_	0.8443	1275	34	1.28	alkanes	/
17.74	811	1326	2-Undecenal^a^	2463-77-6	C_11_H_20_O	0.8678	1367	41	0.74	aldehydes	fruit flavor
18.48	855	1367	2,3-Dihydro-1 h-inden-5-ol^a,c^	1470-94-6	C_9_H_10_O	0.8743	1335	32	1.31	alcohols	/
18.79	875	1384	1-Tetradecene^a,b^	1120-36-1	C_14_H_28_	0.8434	1392	8	0.76	olefins	/
19.08	889	1401	Tetradecane^a,b^	629–59-4	C_14_H_30_	0.9316	1400	1	0.82	alkanes	/
20.71	1024	1494	Dodecyl formate^a,c^	28,303–42-6	C_13_H_26_O_2_	0.8691	1513	19	0.91	esters	/
21.75	1095	1553	Benzoic acid hept-2-yl ester^c,d^	/	C_14_H_20_O_2_	0.8083	1562	9	1.03	esters	/
24.96	1312	1738	7-Methyl-heptadecane^a,c^	20,959–33-5	C_18_H_38_	0.8529	1739	1	0.91	alkanes	/
30.10	1708	2074	Heneicosane^a,d^	629–94-7	C_21_H_44_	0.8652	2100	26	0.80	alkanes	/
30.93	1777	2146	9-Methylheneicosane^a,b,c^	70,475–51-3	C_22_H_46_	0.8570	2130	16	0.78	alkanes	/
31.05	1783	2156	Glutaric acid di(isobutyl) ester^b,d^	/	C_13_H_24_O_4_	0.9455	/	2156	1.69	esters	/
34.27	2069	2517	m-Anisic acid tridec-2-ynyl ester^c,d^	/	C_21_H_30_O_3_	0.8034	2536	19	1.38	esters	/
36.95	2214	2930	Phthalic acid butyl 4-octyl ester^a,d^	/	C_20_H_30_O_4_	0.9034	/	2930	1.64	esters	/

a the content of a compound in Ningxia province was higher than the average value. ^b^ the content of a compound in Gansu province was higher than the average value. ^c^ the content of a compound in Xinjiang province was higher than the average value. ^d^ the content of a compound in Qinghai province was higher than the average value.

#### 3.3.2 Content distribution of VOCs in goji berry

**Fig. 4:**
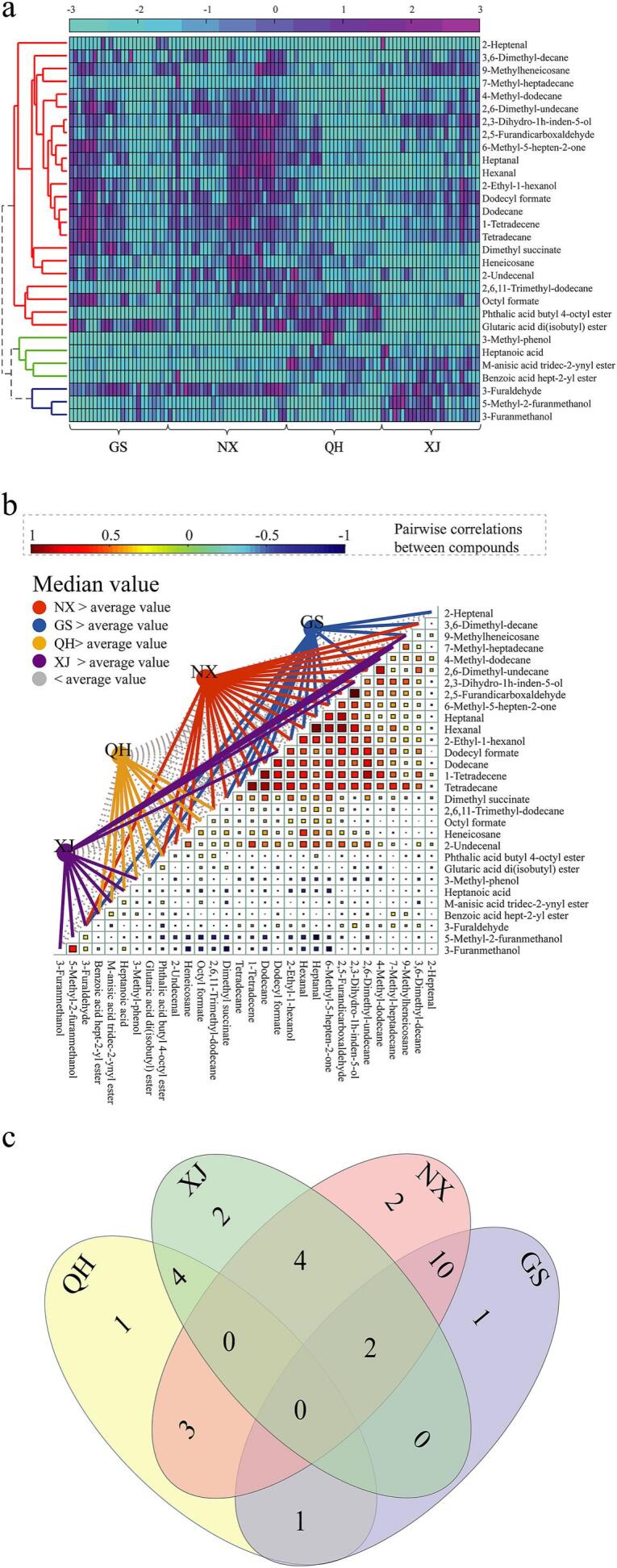
Content distribution (a), correlation (b), and Venn diagram (c) of the 30 identified metabolites. NX, GS, QH, and XJ represent Ningxia, Gansu, Qinghai, and Xinjiang provinces, respectively.

Heatmap analysis was conducted to investigate the content distribution of volatile metabolites in *Goji* berries from different geographical origins, and the results are shown in Fig. 4a. The content distribution of VOCs of *Goji* berries differed in various origins. Ningxia and Gansu provinces accounted for higher contents for a majority of compounds than the other two zones. Fig. 4b shows the results of correlation analysis of the identified compounds and the comparison of compound content in various zones. Notably, a compound with a content larger than the average value was marked with colored lines. A total of 21 compounds had a higher abundance than average values in Ningxia province, and 14 were obtained in Gansu. The 12 compounds from Xinjiang obtained had higher content than the average, 9 compounds expressed higher contents in Qinghai province.

Fig. 4b shows the high correlation of the compounds that displayed higher contents in Ningxia and Gansu. The Venn diagram in Fig. 4c further confirms this finding, where compounds that obtained higher contents than average values was provided. Ningxia and Gansu provinces uniquely shared 10 compounds, which was considerably higher than that between Ningxia versus Xinjiang (4 compounds) and Ningxia versus Qinghai (3 compounds). Fig. 3a shows that the samples from Ningxia and Gansu provinces are difficult to distinguish in the sample clustering results. The Pearson coefficients of the shared compounds between Ningxia and Gansu provinces were further investigated. Nine compounds had an average Pearson coefficient of 0.6961. By contrast, the remaining compound, 3-furaldehyde, attained an average Pearson coefficient of 0.1623. The results of relationship among identified compounds in Fig. 4**b** and **c** were consistent with sample clustering results in Fig. 3. In conclusion, the identified compounds can briefly reflect the sample clustering results and may be used as a subset for geographical discrimination.

**Fig. 5:**
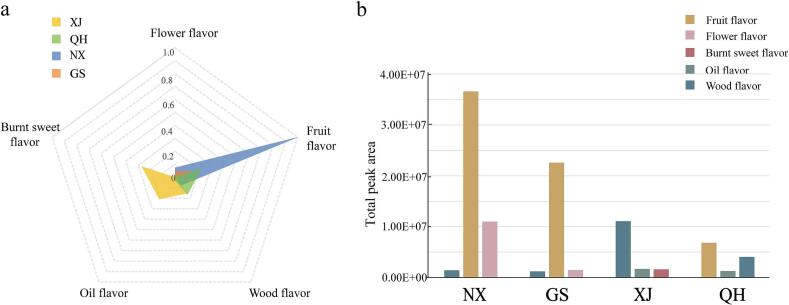
Flavor distribution analysis of *Goji* from different cultivated zones. (a), Radar diagram representing the proportions of the four origins in the five flavors (b), total peak area of different flavors in the four regions. NX, GS, QH, and XJ represent Ningxia, Gansu, Qinghai, and Xinjiang provinces, respectively.

Flavor analysis was performed based on the identified compounds. Eleven compounds were characterized with an aroma, and five types of flavors (Table 1), including flower, fruit, oil, woody, and burnt sweet flavors, were obtained. Fig. 5a shows a radar diagram revealing the peak area proportion of the four areas in the five flavors. Ningxia, Gansu, and Qinghai accounted for the largest proportions of fruit flavor and Xinjiang had the largest proportion of burnt sweet flavor. The bar chart in Fig. 5b illustrates the total peak area of the compounds for specific flavors and the different flavors for each region. The chart reveals the presence of three main flavors in each region. Ningxia and Gansu provinces mainly have fruit, flower, (Lu et al., 2017) and woody flavors. Xinjiang has oil, wood, and burnt sweet flavors. Qinghai province has fruit, oil, and wood flavors. Both images in Fig. 5 reveal the highest contents of fruit flavor in Ningxia province.

The identified 30 compounds across 8 categories collectively define the unique flavor profile of *Goji* berries. Notably, aldehydes, esters, and alcohols dominate the aroma-active compounds, contributing fruity and flower flavors. (Mou et al., 2025) These compounds likely act synergistically to establish the signature flavor of Ningxia berries, characterized by pronounced fruity intensity. Phenolic compounds (3-methylphenol) may introduce subtle woody undertones, (Feng et al., 2016; Jordão, Correia, Botelho, Ortega-Heras, & González-SanJosé, 2025) balancing the overall complexity of the flavor profile. Geographical proximity and climatic similarities between Gansu and Ningxia make their berries difficult to distinguish, with both exhibiting comparable flavor compositions dominated by fruity flavor. However, Gansu berries generally show lower concentrations of flavor compounds compared to their Ningxia counterparts. In Xinjiang samples, the distinctive burnt sweet flavors likely originates from Maillard reaction-derived compounds (furan derivatives 5-methyl-2-furanmethanol) formed during prolonged sun-drying under intense solar exposure. (Boateng & Yang, 2021; Han et al., 2025).

#### 3.3.3 Prediction of goji berry geographical location using different discriminant models

**Fig. 6:**
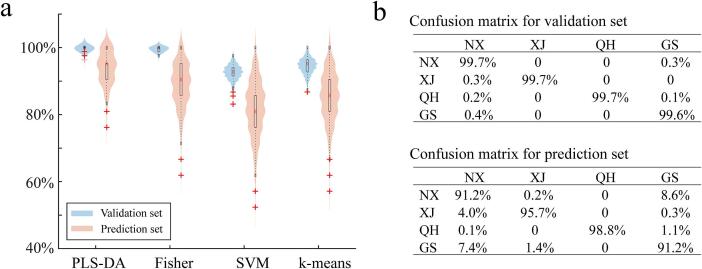
(a) Characterization of the accuracy distribution of each model. (b) Confusion matrix of PLS-DA. NX, GS, QH, and XJ represent Ningxia, Gansu, Qinghai, and Xinjiang provinces, respectively.

Based on the identified 30 compounds, geographical discrimination models were constructed and compared in detail. Notably, model construction involved the original peak area of each compound. Four classic chemometric methods were introduced, PLS-DA, Fisher discrimination, SVM, and the one based on *k*-means clustering. For each clustering method, Monte–Carlo simulation was employed. A total of 80 % of the samples in each zone were allotted to a validation set for model construction, and the remaining 20 % were used for prediction. After 10,000 simulations, the accuracy of each model was statistically analyzed (Fig. 6a**)**. The PLS-DA model obtained the best performance by providing the highest values for validation and prediction sets. The averages validation and prediction were 99.7 % and 94.2 %, respectively. Slightly lower values was found for *k*-means, whose validation and prediction values reached 94.5 % and 85.4 %, respectively. The Fisher discrimination and SVM methods provided considerably lower accuracy values (Fig. 6a), especially for the prediction accuracy. Thus, the PLS-DA model may be used for geographical discrimination based on identified compounds.

The confusion matrix of the PLS-DA was further investigated, and the results are indicated in Fig. 6b. Most of the samples were accurately classified in the validation set, as the accuracy for each zone can reach up to 99.6 %. The prediction accuracy for each zone was a slightly lower. Samples from Xinjiang and Qinghai provinces displayed slightly higher accuracy values, with accuracy values of 95.7 % and 98.8 %, respectively. The values for Ningxia and Gansu both were 91.2 %. The prediction accuracy was consistent with the geographical locations, given that the climate of Qinghai and Xinjiang provinces differ from those of Gansu and Ningxia. The cultivated zones in Ningxia were close to a majority of collected samples from Gansu. A total of 8.6 % of samples from the Ningxia province were inaccurately arranged into Gansu. On the contrary, 1.4 % samples from Gansu province was inaccurately classified under Xinjiang, possibly because some cultivate zones in Gansu were closer to Xinjiang. Notably, samples from various zones can be separated satisfactorily using the developed strategy. The abovementioned results indicate that the combination of HS-SPME-GCMS with AntDAS-GCMS can be used to comprehensively characterize VOCs in *Goji* berries for flavor compound identification and accurate tracing of geographical zones of *Goji* berry.

## 4 Conclusion

In this study, we proposed a new strategy for the characterization of VOCs of *Goji* berries using HS-SPME-GCMS combined with AntDAS-GCMS. A total of 275 compounds were screened, and 30 were identified. The identified compounds can be used for the construction of a chemometric model for the accurate tracing of the origins of *Goji* berries from various cultivation zones in the northwest zones of China. A comparison of different chemometric methods, including PLS-DA, Fisher discrimination, SVM, and *k*-means, indicated that PLS-DA can provide the best performance for geographical discrimination. The developed strategy offers an alternative solution for the analysis of VOCs in complex samples.

## CRediT authorship contribution statement

**Lu Han:** Writing – original draft, Visualization, Investigation. **Wan-Ting Zou:** Writing – review & editing. **Wen-Xin Wang:** Writing – review & editing. **Long-He Wang:** Writing – review & editing. **Ping-Ping Liu:** Writing – review & editing. **Li-Hua Tang:** Writing – review & editing. **Yi Lv:** Writing – review & editing. **Yong-Jie Yu:** Writing – review & editing, Methodology. **Yuanbin She:** Writing – review & editing.

## Declaration of competing interest

The authors declare that they have no known competing financial interests or personal relationships that could have appeared to influence the work reported in this paper.

## Data Availability

Data will be made available on request.

## Appendix B Supplementary data

Supplementary material Image 1

## References

[bb0005] Boateng I.D., Yang X.-M. (2021). Thermal and non-thermal processing affect Maillard reaction products, flavor, and phytochemical profiles of Ginkgo biloba seed. Food Bioscience.

[bb0010] Cao Y.-L., Li Y.-L., Fan Y.-F., Li Z., Yoshida K., Wang J.-Y., Liu Z.-J. (2021). Wolfberry genomes and the evolution of Lycium (Solanaceae). Communications Biology.

[bb0015] Cheng H., Wu W., Chen J., Pan H., Xu E., Chen S., Chen J. (2022). Establishment of anthocyanin fingerprint in black wolfberry fruit for quality and geographical origin identification. Lwt.

[bb0020] Dong F., Hao J., Luo R., Zhang Z., Wang S., Wu K., Liu M. (2022). Identification of the proximate geographical origin of wolfberries by two-dimensional correlation spectroscopy combined with deep learning. Computers and Electronics in Agriculture.

[bb0025] Fan X., Xu Z., Zhang H., Liu D., Yang Q., Tao Q., Lu H. (2022). Fully automatic resolution of untargeted GC-MS data with deep learning assistance. Talanta.

[bb0030] Feng J., Jiang J., Yang Z., Su Q., Wang K., Xu J. (2016). Characterization of depolymerized lignin and renewable phenolic compounds from liquefied waste biomass. RSC Advances.

[bb0035] Gong H., Rehman F., Li Z., Liu J., Yang T., Liu J., Wang Y. (2022). Discrimination of geographical origins of wolfberry (Lycium barbarum L.) fruits using stable isotopes, earth elements, free amino acids, and saccharides. Journal of Agricultural and Food Chemistry.

[bb0040] Han X., Gao X., Yu M., Xu R., Song H., Pan W., Huang Z. (2025). Sensory directed analysis for key odor-active compounds in by-products flavorings enhanced by enzymatic hydrolysis and Maillard reaction. Journal of Food Composition and Analysis.

[bb0045] He J., Wang T., Yan H., Guo S., Hu K., Yang X., Duan J. (2023). Intelligent identification method of geographic origin for Chinese wolfberries based on color space transformation and texture morphological features. Foods.

[bb0050] Jiang X., Liu Q., Yan L., Cao X., Chen Y., Wei Y., Xing H. (2024). Hyperspectral imaging combined with spectral-imagery feature fusion convolutional neural network to discriminate different geographical origins of wolfberries. Journal of Food Composition and Analysis.

[bb0055] Jordão A.M., Correia A.C., Botelho R.V., Ortega-Heras M., González-SanJosé M.L. (2025). Phenolic content, volatile composition and sensory profile of red wines macerated with toasted woods from different south American botanical species. Journal of Food Composition and Analysis.

[bb0060] Li Q., Yu X., Xu L., Gao J.-M. (2017). Novel method for the producing area identification of Zhongning goji berries by electronic nose. Food Chemistry.

[bb0065] Li Y., Wang X., Sa Y., Li L., Wang W., Yang L., Ma X. (2024). A comparative UHPLC-QTOF-MS/MS-based metabolomics approach reveals the metabolite profiling of wolfberry sourced from different geographical origins. Food Chemistry: X.

[bb0070] Liang J., Wu H., Lu M., Li Y. (2024). HS-SPME-GC-MS untargeted metabolomics reveals key volatile compound changes during Liupao tea fermentation. Food Chemistry: X.

[bb0075] Liu H., Rao H., Zhou H., Li J., Li H., Guo J., Du X. (2024). A novel top-down strategy for in situ construction of vertically oriented hexagonal NiCr LDHs nanosheet arrays with intercalated sulfate ions on Nichrome fiber for selective solid-phase microextraction of phenolic compounds in water samples. Analytica Chimica Acta.

[bb0080] Lu J., Li H., Quan J., An W., Zhao J., Xi W. (2017). Identification of characteristic aroma volatiles of Ningxia goji berries (Lycium barbarum L.) and their developmental changes. International Journal of Food Properties.

[bb0085] Ma G.-M., Wang J.-N., Wang X.-C., Ma F.-L., Wang W.-X., Li S.-F., She Y. (2024). AntDAS-GCMS: A new comprehensive data analysis platform for GC–MS-based untargeted metabolomics with the advantage of addressing the time shift problem. Analytical Chemistry.

[bb0090] Ma M., Chen Z., Huang B., Chen X., Liu H., Peng Z., Wu D. (2024). Characterizing the key aroma compounds of barley malt from different origins using GC-E-nose, HS-SPME-GC-MS, and HS-GC-IMS. Food Bioscience.

[bb0095] Ma R.-H., Zhang X.-X., Ni Z.-J., Thakur K., Wang W., Yan Y.-M., Wei Z.-J. (2022). Lycium barbarum (goji) as functional food: A review of its nutrition, phytochemical structure, biological features, and food industry prospects. Critical Reviews in Food Science and Nutrition.

[bb0100] Ma X.-L., Wang X.-C., Zhang J.-N., Liu J.-N., Ma M.-H., Ma F.-L., She Y. (2023). A study of flavor variations during the flaxseed roasting procedure by developed real-time SPME GC–MS coupled with chemometrics. Food Chemistry.

[bb0105] Mou Z., Cai X., Liu J., Deng R., Liu Z., Fan R., Luo A. (2025). Elucidation of the key aroma compounds of floral and fruity aroma in sauce-flavored baijiu by pervaporative membrane separation, GC-IMS, GC-MS, and aroma omission studies. Food Bioscience.

[bb0110] Peng Q., Huang J., Li S., Massou B.B., Chen Z., Zhu Q., Xie G. (2024). Comprehensive origin authentication of wolfberry pulp (Lycium barbarum L.) using multimodal sensory analysis and chemometrics. Industrial Crops and Products.

[bb0115] Popović-Djordjević J., Kostić A.Ž., Kamiloglu S., Tomas M., Mićanović N., Capanoglu E. (2023). Chemical composition, nutritional and health related properties of the medlar (Mespilus germanica L.): From medieval glory to underutilized fruit. Phytochemistry Reviews.

[bb0120] Smirnov A., Jia W., Walker D.I., Jones D.P., Du X. (2017). ADAP-GC 3.2: Graphical software tool for efficient spectral deconvolution of gas chromatography–high-resolution mass spectrometry metabolomics data. Journal of Proteome Research.

[bb0125] Stein S.E., Scott D.R. (1994). Optimization and testing of mass spectral library search algorithms for compound identification. Journal of the American Society for Mass Spectrometry.

[bb0130] Tauler R. (2020). Multivariate curve resolution of multiway data using the multilinearity constraint. Journal of Chemometrics.

[bb0135] Xu Y., Yao L., Wang Y., Shen J., Chen D., Feng T. (2024). Comparative analysis of the aromatic profiles of citri sarcodactylis fructus from various geographical regions using GC-IMS, GC-MS, and sensory evaluation. Food Bioscience.

[bb0140] Zhang Y.-Y., Zhang Q., Zhang Y.-M., Wang W.-W., Zhang L., Yu Y.-J., She Y. (2020). A comprehensive automatic data analysis strategy for gas chromatography-mass spectrometry based untargeted metabolomics. Journal of Chromatography A.

[bb0145] Zhao J.-J., Guo X.-M., Wang X.-C., Zhang Y., Ma X.-L., Ma M.-H., She Y.-B. (2022). A chemometric strategy to automatically screen selected ion monitoring ions for gas chromatography–mass spectrometry-based pseudotargeted metabolomics. Journal of Chromatography A.

[bb0150] Zhou L., Guan Y., Yao J., Zhao M., Fu H., Liu J., Marchioni E. (2023). Identification and discrimination of lilii bulbus origins based on lipidomics using UHPLC–QE-Orbitrap/MS/MS combined with chemometrics analysis. Journal of Food Composition and Analysis.

[bb0155] Zhou Y., Wang D., Duan H., Zhou S., Guo J., Yan W. (2023). Detection and analysis of volatile flavor compounds in different varieties and origins of goji berries using HS-GC-IMS. Lwt.

