# Supplementary material for: Comprehensive characterization of flavor compounds in goji berry by HS-SPME-GCMS combined with AntDAS-GCMS for geographical discrimination
Source: Food Chem X. 2025 Jun 4;29:102626. doi: 10.1016/j.fochx.2025.102626 (PMC12182799; doi:10.1016/j.fochx.2025.102626)
Supplement: Supplementary file 1 — Supplementary material [file mmc1.docx]

**Supporting Information**

**Comprehensive characterization of flavor compounds in *Goji* berry by HS-SPME-GCMS combined with AntDAS-GCMS for geographical discrimination**

Lu Han^a,e^, Wan-Ting Zou^a,e^, Wen-Xin Wang^a,e^, Long-He Wang^a,e^, Ping-Ping Liu^b^, Li-Hua Tang^c,^*, Yi Lv^c^, Yong-Jie Yu^a,e^*, Yuanbin She^d^

*^a^ College of Pharmacy, Ningxia Medical University, Yinchuan 750004, China*

*^b^ Zhengzhou Tobacco Research Institute of CNTC, Zhengzhou 450001, China*

*^c^ Ningxia Food Testing Research Institute, Yinchuan 750004, China*

*^d^ College of Chemical Engineering, Zhejiang University of Technology, Hangzhou 310032, China*

*^e^ Key Laboratory of Ningxia Minority Medicine Modernization, Ministry of Education, Yinchuan 750004, China*

*^*^Corresponding author*

E-mail address: [yongjie.yu@163.com](mailto:yongjie.yu@163.com); tanglihua532@163.com


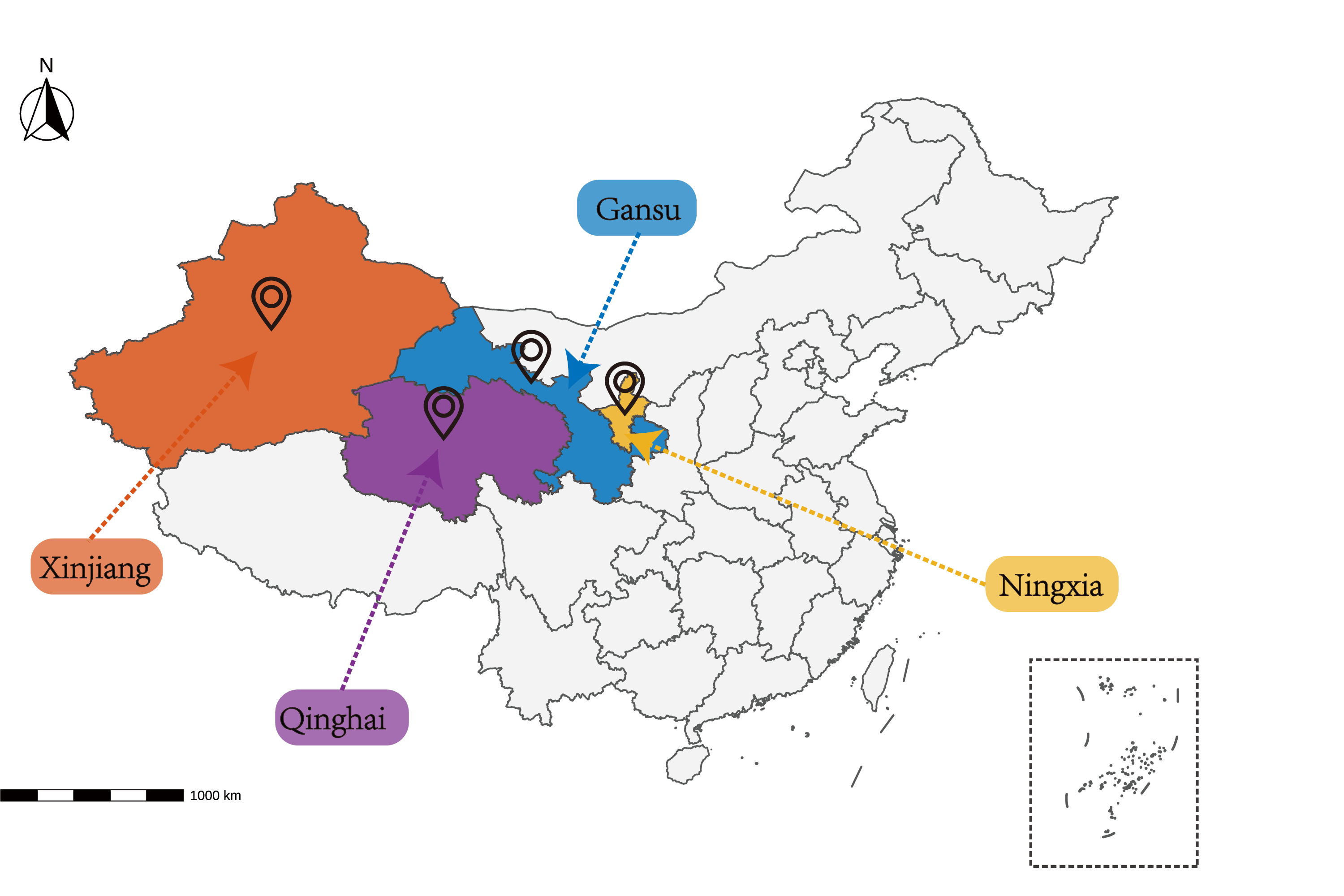


**Figure S1.** A Schematic diagram of the geographical distribution of the four different *Goji* berry producing areas in China.


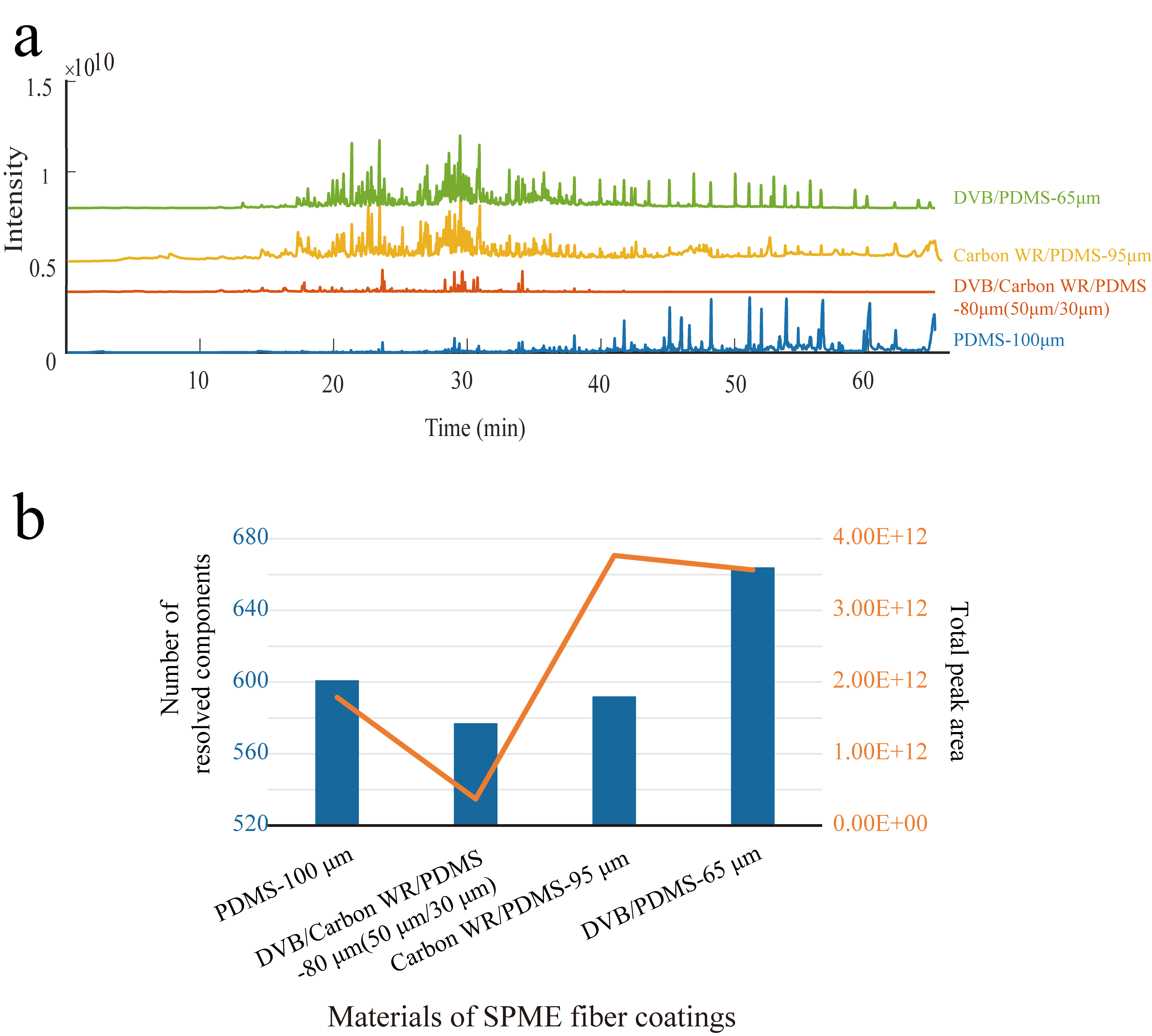


**Figure S2.** Optimization of materials on the fiber of SPME. (a) TICs corresponding to different types of fibers. (b) Number of resolved components and total peak area of components.


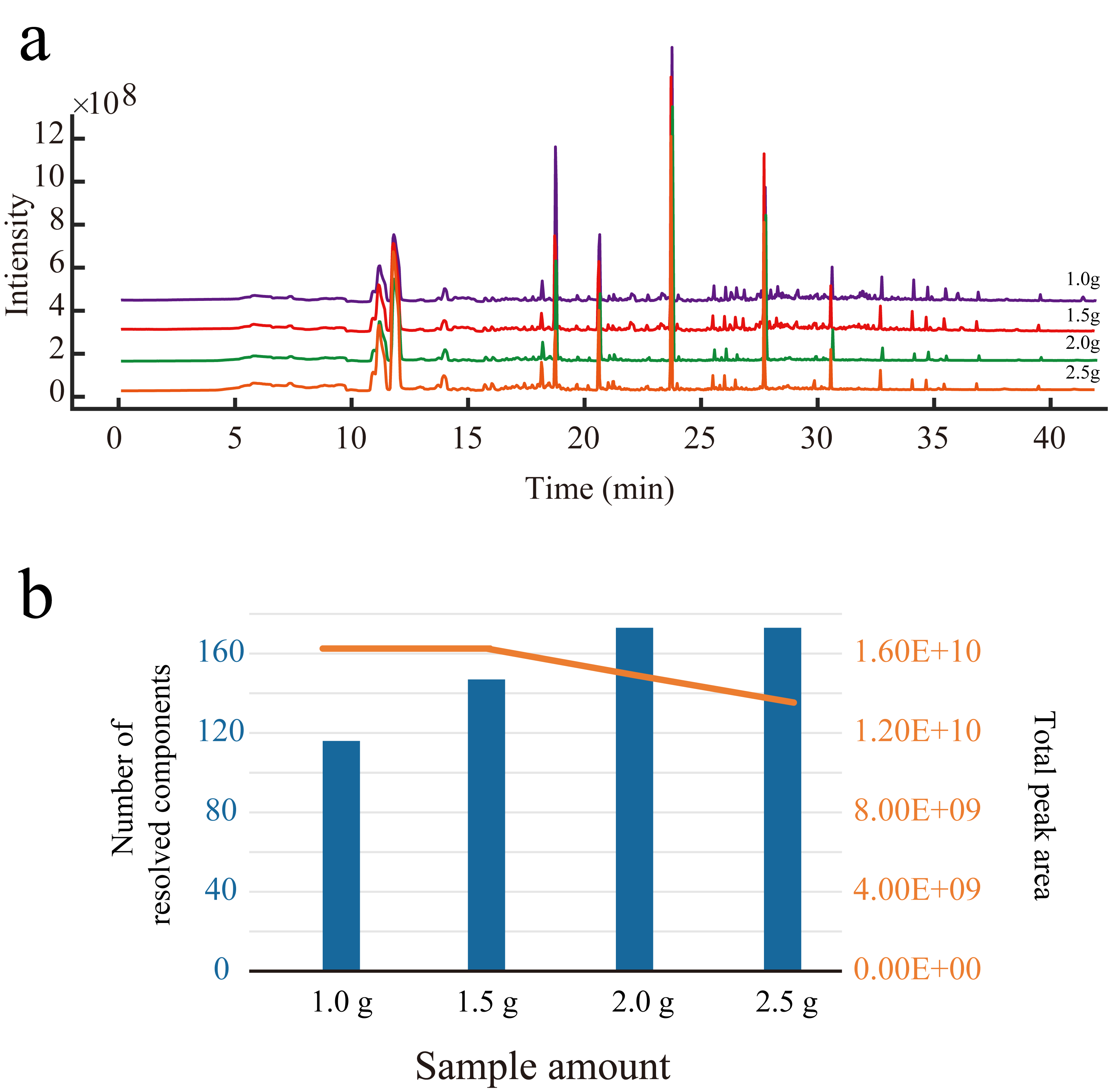


**Figure S3.** Optimization of sample amount. (a) TICs of different sample amounts. (b) Number of resolved components and total peak area of components.

**
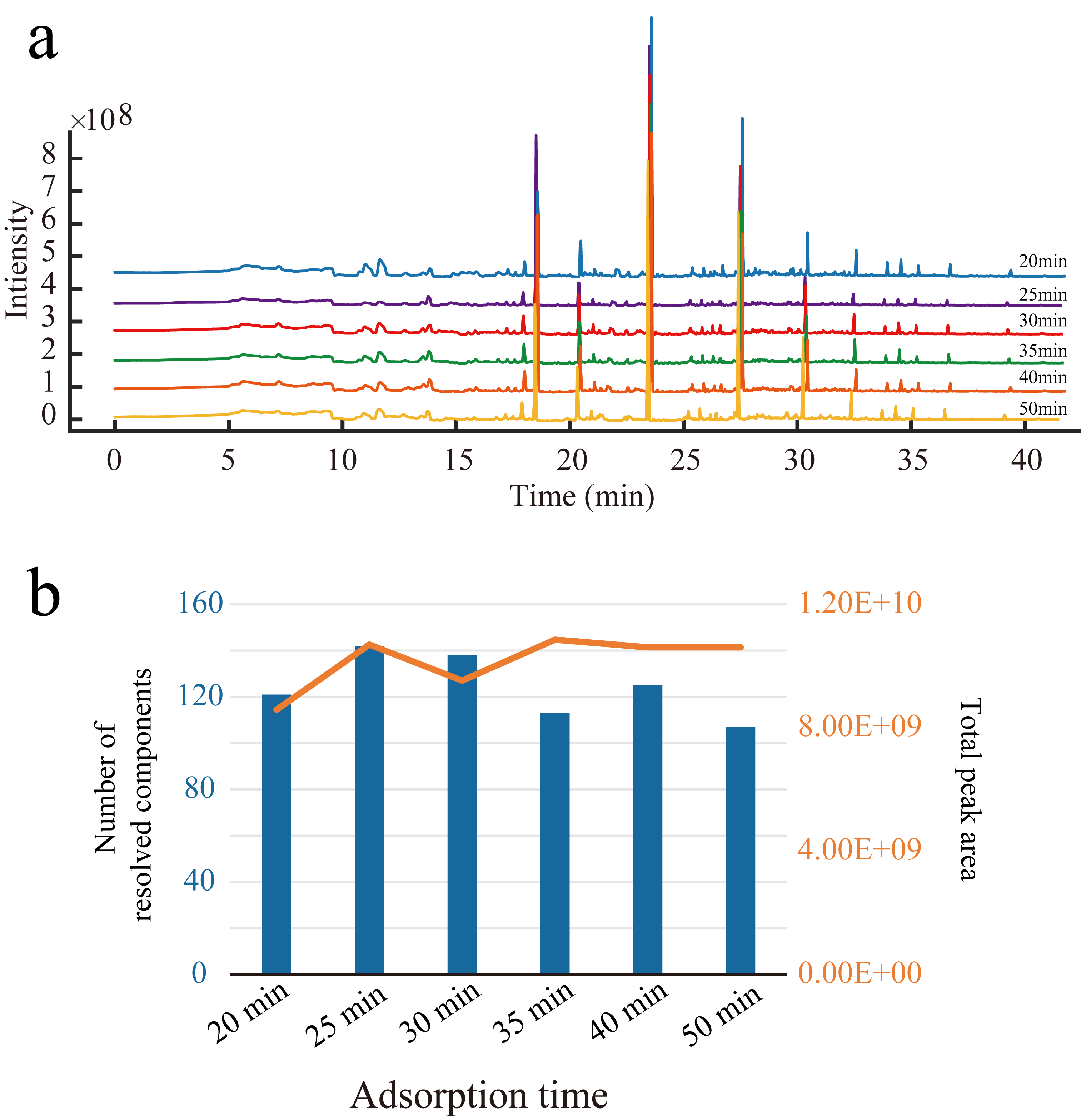
**

**Figure S4.** Optimization of adsorption time. (a) TICs of various adsorption times. (b) Number of resolved components and total peak area of components.


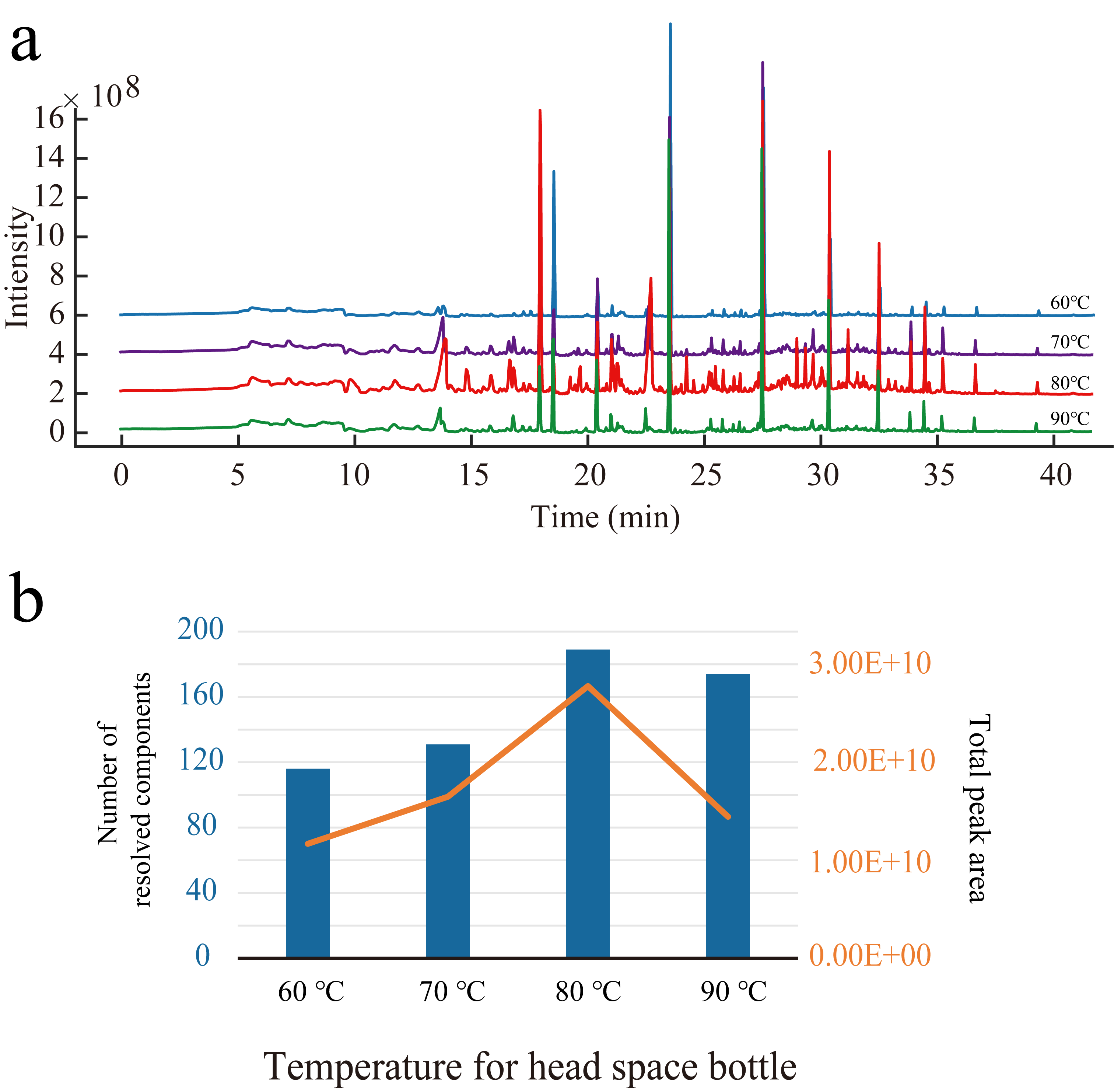


**Figure S5.** Optimization of heating temperature for *Goji* samples. (a) TICs of different heating temperatures. (b) Number of resolved components and total peak area of components.

**Table S1**. Geographical origin of the 104 *Goji* berries samples

| Number | Sample name | Origin |
| --- | --- | --- |
| 1 | NX-1 | Zhouta , Zhongwei, Ningxia |
| 2 | NX-2 | Zhouta , Zhongwei, Ningxia |
| 3 | NX-3 | Zhouta , Zhongwei, Ningxia |
| 4 | NX-4 | Zhouta , Zhongwei, Ningxia |
| 5 | NX-5 | Zhouta , Zhongwei, Ningxia |
| 6 | NX-6 | Zhouta , Zhongwei, Ningxia |
| 7 | NX-7 | Zhouta , Zhongwei, Ningxia |
| 8 | NX-8 | Zhouta , Zhongwei, Ningxia |
| 9 | NX-9 | Zhouta , Zhongwei, Ningxia |
| 10 | NX-10 | Zhouta , Zhongwei, Ningxia |
| 11 | NX-11 | Zhouta , Zhongwei, Ningxia |
| 12 | NX-12 | Zhouta , Zhongwei, Ningxia |
| 13 | NX-13 | Zhouta , Zhongwei, Ningxia |
| 14 | NX-14 | Zhouta , Zhongwei, Ningxia |
| 15 | NX-15 | Zhouta , Zhongwei, Ningxia |
| 16 | NX-16 | Zhouta , Zhongwei, Ningxia |
| 17 | NX-17 | Zhouta , Zhongwei, Ningxia |
| 18 | NX-18 | Zhouta , Zhongwei, Ningxia |
| 19 | NX-19 | Zhouta , Zhongwei, Ningxia |
| 20 | NX-20 | Zhouta , Zhongwei, Ningxia |
| 21 | NX-21 | Zhouta , Zhongwei, Ningxia |
| 22 | NX-22 | Zhouta , Zhongwei, Ningxia |
| 23 | NX-23 | Zhouta , Zhongwei, Ningxia |
| 24 | NX-24 | Zhouta , Zhongwei, Ningxia |
| 25 | NX-25 | Zhouta , Zhongwei, Ningxia |
| 26 | NX-26 | Zhouta , Zhongwei, Ningxia |
| 27 | NX-27 | Zhouta , Zhongwei, Ningxia |
| 28 | NX-28 | Zhouta , Zhongwei, Ningxia |
| 29 | NX-29 | Zhouta , Zhongwei, Ningxia |
| 30 | NX-30 | Zhouta , Zhongwei, Ningxia |
| 31 | GS-1 | Liu Zhaike, Baiyin, Gansu |
| 32 | GS-2 | Liu Zhaike, Baiyin, Gansu |
| 33 | GS-3 | Liu Zhaike, Baiyin, Gansu |
| 34 | GS-4 | Liu Zhaike, Baiyin, Gansu |
| 35 | GS-5 | Liu Zhaike, Baiyin, Gansu |
| 36 | GS-6 | Liu Zhaike, Baiyin, Gansu |
| 37 | GS-7 | Liu Zhaike, Baiyin, Gansu |
| 38 | GS-8 | Liu Zhaike, Baiyin, Gansu |
| 39 | GS-9 | Liu Zhaike, Baiyin, Gansu |
| 40 | GS-10 | Liu Zhaike, Baiyin, Gansu |
| 41 | GS-11 | Liu Zhaike, Baiyin, Gansu |
| 42 | GS-12 | Liu Zhaike, Baiyin, Gansu |
| 43 | GS-13 | Liu Zhaike, Baiyin, Gansu |
| 44 | GS-14 | Liu Zhaike, Baiyin, Gansu |
| 45 | GS-15 | Liu Zhaike, Baiyin, Gansu |
| 46 | GS-16 | Liu Zhaike, Baiyin, Gansu |
| 47 | GS-17 | Liu Zhaike, Baiyin, Gansu |
| 48 | GS-18 | Liu Zhaike, Baiyin, Gansu |
| 49 | GS-19 | Liu Zhaike, Baiyin, Gansu |
| 50 | GS-20 | Liu Zhaike, Baiyin, Gansu |
| 51 | GS-21 | Liu Zhaike, Baiyin, Gansu |
| 52 | GS-22 | Liu Zhaike, Baiyin, Gansu |
| 53 | GS-23 | Liu Zhaike, Baiyin, Gansu |
| 54 | GS-24 | Liu Zhaike, Baiyin, Gansu |
| 55 | GS-25 | Liu Zhaike, Baiyin, Gansu |
| 56 | QH-1 | Delingha, Haixi, Qinghai |
| 57 | QH-2 | Delingha, Haixi, Qinghai |
| 58 | QH-3 | Delingha, Haixi, Qinghai |
| 59 | QH-4 | Delingha, Haixi, Qinghai |
| 60 | QH-5 | Delingha, Haixi, Qinghai |
| 61 | QH-6 | Delingha, Haixi, Qinghai |
| 62 | QH-7 | Delingha, Haixi, Qinghai |
| 63 | QH-8 | Delingha, Haixi, Qinghai |
| 64 | QH-9 | Delingha, Haixi, Qinghai |
| 65 | QH-10 | Delingha, Haixi, Qinghai |
| 66 | QH-11 | Delingha, Haixi, Qinghai |
| 67 | QH-12 | Delingha, Haixi, Qinghai |
| 68 | QH-13 | Delingha, Haixi, Qinghai |
| 69 | QH-14 | Delingha, Haixi, Qinghai |
| 70 | QH-15 | Delingha, Haixi, Qinghai |
| 71 | QH-16 | Delingha, Haixi, Qinghai |
| 72 | QH-17 | Delingha, Haixi, Qinghai |
| 73 | QH-18 | Delingha, Haixi, Qinghai |
| 74 | QH-19 | Delingha, Haixi, Qinghai |
| 75 | QH-20 | Delingha, Haixi, Qinghai |
| 76 | QH-21 | Delingha, Haixi, Qinghai |
| 77 | QH-22 | Delingha, Haixi, Qinghai |
| 78 | QH-23 | Delingha, Haixi, Qinghai |
| 79 | QH-24 | Delingha, Haixi, Qinghai |
| 80 | XJ-1 | Jinghe, Bortala, Xinjiang |
| 81 | XJ-2 | Jinghe, Bortala, Xinjiang |
| 82 | XJ-3 | Jinghe, Bortala, Xinjiang |
| 83 | XJ-4 | Jinghe, Bortala, Xinjiang |
| 84 | XJ-5 | Jinghe, Bortala, Xinjiang |
| 85 | XJ-6 | Jinghe, Bortala, Xinjiang |
| 86 | XJ-7 | Jinghe, Bortala, Xinjiang |
| 87 | XJ-8 | Jinghe, Bortala, Xinjiang |
| 88 | XJ-9 | Jinghe, Bortala, Xinjiang |
| 89 | XJ-10 | Jinghe, Bortala, Xinjiang |
| 90 | XJ-11 | Jinghe, Bortala, Xinjiang |
| 91 | XJ-12 | Jinghe, Bortala, Xinjiang |
| 92 | XJ-13 | Jinghe, Bortala, Xinjiang |
| 93 | XJ-14 | Jinghe, Bortala, Xinjiang |
| 94 | XJ-15 | Jinghe, Bortala, Xinjiang |
| 95 | XJ-16 | Jinghe, Bortala, Xinjiang |
| 96 | XJ-17 | Jinghe, Bortala, Xinjiang |
| 97 | XJ-18 | Jinghe, Bortala, Xinjiang |
| 98 | XJ-19 | Jinghe, Bortala, Xinjiang |
| 99 | XJ-20 | Jinghe, Bortala, Xinjiang |
| 100 | XJ-21 | Jinghe, Bortala, Xinjiang |
| 101 | XJ-22 | Jinghe, Bortala, Xinjiang |
| 102 | XJ-23 | Jinghe, Bortala, Xinjiang |
| 103 | XJ-24 | Jinghe, Bortala, Xinjiang |
| 104 | XJ-25 | Jinghe, Bortala, Xinjiang |
